# Supplementary figures and images for: Characterization of genes and alleles involved in the control of flowering time in grapevine
Source: PLoS One. 2019 Jul 3;14(7):e0214703. doi: 10.1371/journal.pone.0214703 (PMC6608932; doi:10.1371/journal.pone.0214703)

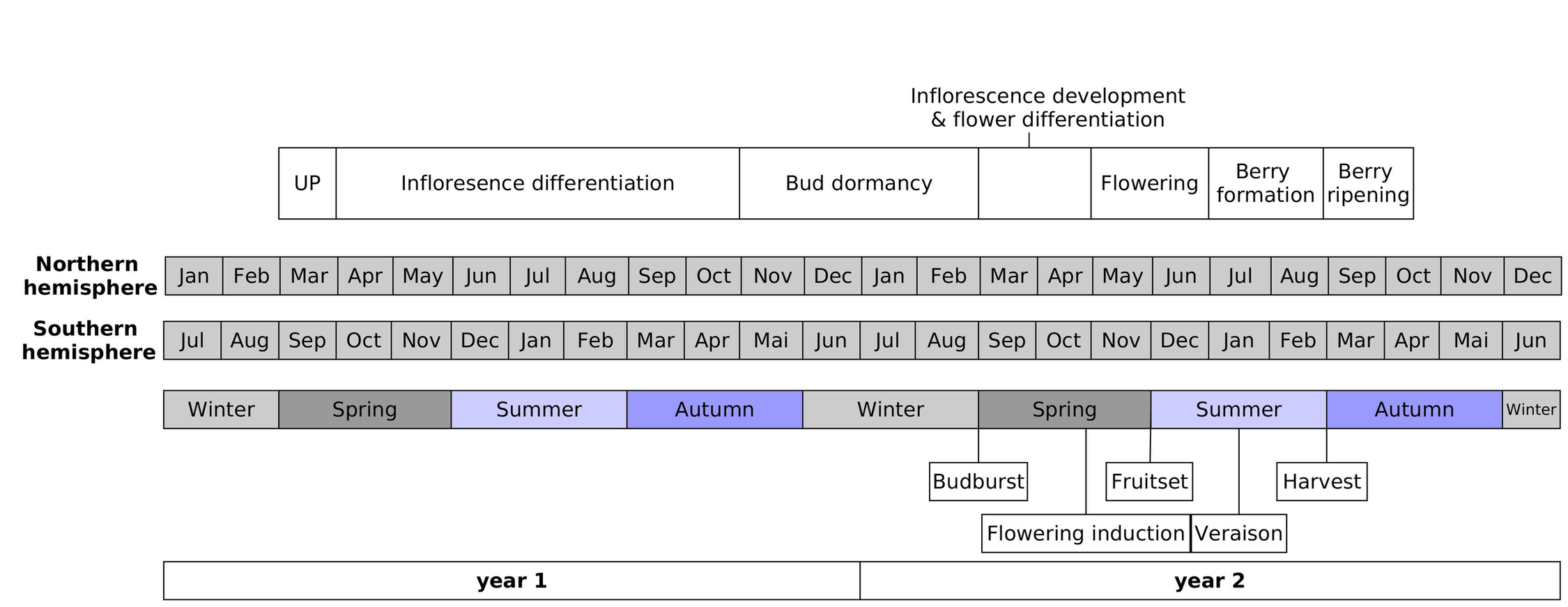

Supplement: S1 Fig — Illustration of the reproductive developmental cycle of grapevine showing the stages of flowering and berry development (according to 1.). UP: uncommitted primordia. (TIF) [file pone.0214703.s001.tif]

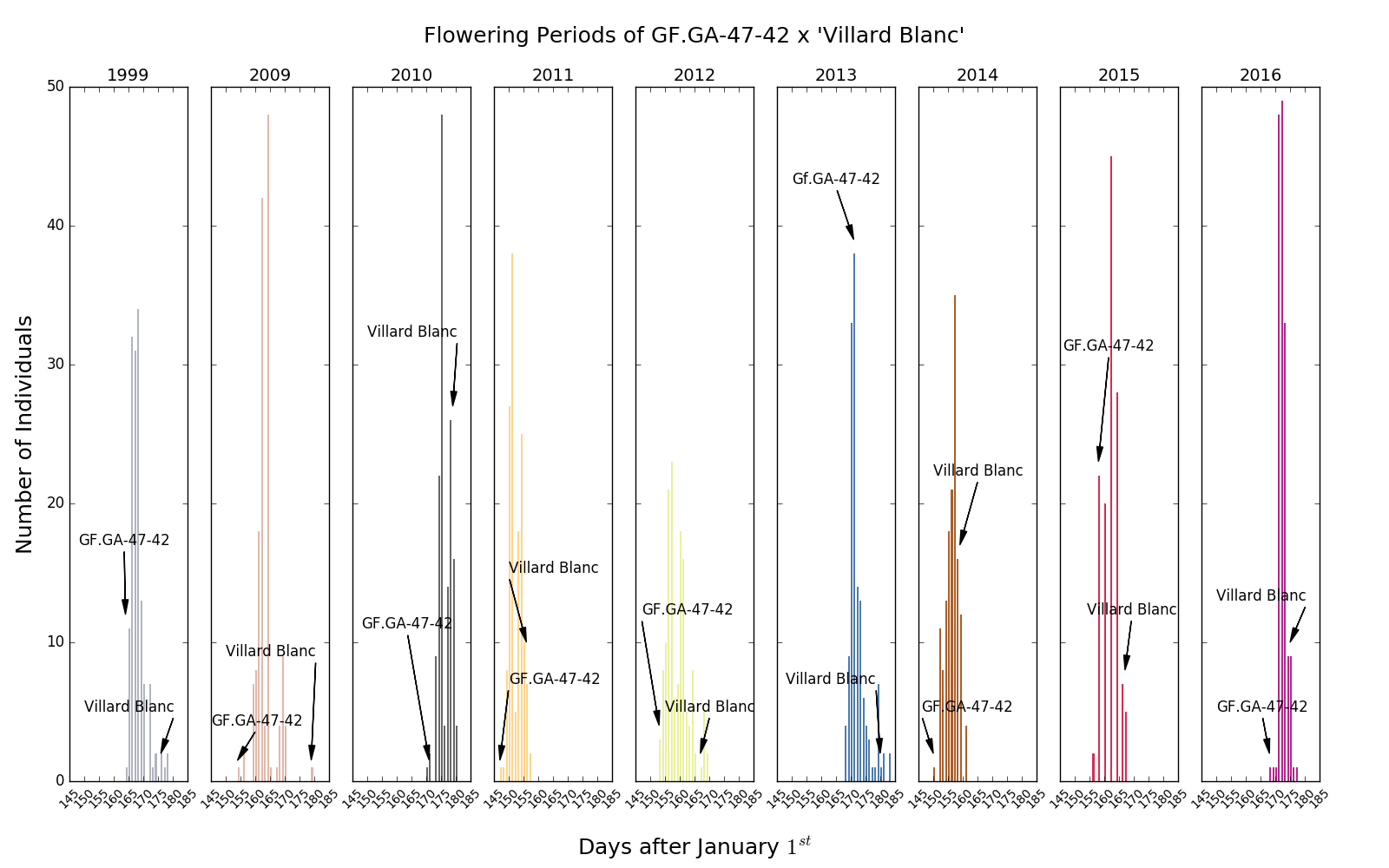

Supplement: S2 Fig — Flowering periods in days after January 1st in the population GF.GA-47-42 x ‘Villard Blanc’ in the years 1999 and 200910–2016 with flowering dates of GF.GA-47-42 and ‘Villard Blanc’ highlighted insofar available. (TIF) [file pone.0214703.s002.tif]

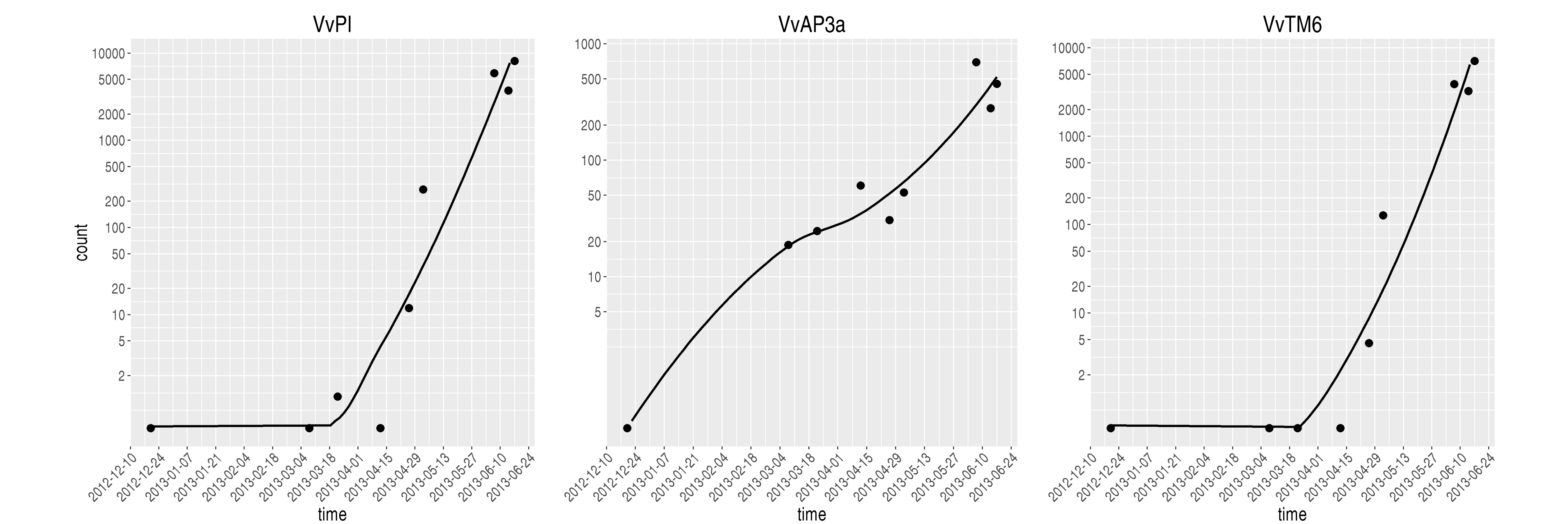

Supplement: S3 Fig — Expression profile of the three B-class floral homeotic genes VvAP3, VvTM6 and VvPI over consecutive developmental stages of bud- and inflorescence development in GF.GA-47-42. The last three time points refer to developing stages of visible inflorescence structures. (TIF) [file pone.0214703.s003.tif]
